# Рrospective multicenter study of treatment efficacy, safety, and quality of life in a large cohort of patients with inborn errors of immunity receiving subcutaneous immunoglobulin by the rapid push method

**DOI:** 10.3389/fimmu.2025.1598491

**Published:** 2025-07-22

**Authors:** Asmik Avedova, Elena Deripapa, Yulia Rodina, Anna Mukhina, Elena Latysheva, Daria Yukhacheva, Vasily Burlakov, Nellie Kan, Daria Bogdanova, Anna Moiseeva, Natalia Kuzmenko, Zoya Nesterenko, Ekaterina Deordieva, Anna Ogneva, Victoria Bludova, Anna Roppelt, Daria Fomina, Natalia Zinovieva, Yulia Sevostyanova, Linara Kalmeteva, Dilara Prolygina, Lyudmila Barycheva, Olga Selezneva, Natalya Shakhova, Olga Laba, Elena Vlasova, Alla Gorenkova, Elena Timofeeva, Olga Trusova, Marina Guseva, Natalya Yudina, Evgeniya Grevtseva, Asya Ibisheva, Zema Bambaeva, Dina Mashkovskaya, Svetlana Isakova, Almazia Shakirova, Ekaterina Selina, Tatyana Shilova, Elena Zubova, Iman Khabaeva, Ekaterina Kitova, Anastasia Mandzhieva, Svetlana Starikova, Tatyana Pavlova, Elvira Tyulyakova, Pavel Levin, Nikolay Grachev, Anna Shcherbina

**Affiliations:** ^1^ Immunology Department, Dmitry Rogachev National Medical Research Center of Pediatric Hematology, Oncology and Immunology, Moscow, Russia; ^2^ Immunopathology Department, National Research Center Institute of Immunology Federal Medical and Biological Agency (FMBA), Moscow, Russia; ^3^ Moscow Center of Allergy and Immunology, Clinical City Hospital 52 Ministry of Healthcare of Moscow, Moscow, Russia; ^4^ Immunology Department, Children’s City Clinical Hospital №9, Moscow, Russia; ^5^ Immunology Department, Republican Children’s Clinical Hospital of the Ministry of Health of the Republic Bashkortostan, Ufa, Russia; ^6^ Immunology Department, Stavropol State Medical University of the Ministry of Health of Russia, Stavropol, Russia; ^7^ Immunology Department, Regional Children’s Clinical Hospital, Rostov-on-Don, Russia; ^8^ Immunology Department, Altai State Medical University of the Ministry of Health of Russia, Barnaul, Russia; ^9^ Immunology Department, Regional Children’s Clinical Hospital, Yaroslavl, Russia; ^10^ Immunology Department, Regional Children’s Clinical Hospital, Yekaterinburg, Russia; ^11^ Immunology Department, Arkhangelsk Regional Children’s Clinical Hospital named after P.G. Vyzhletsova, Arkhangelsk, Russia; ^12^ Immunology Department, Nizhny Novgorod Regional Children’s Clinical Hospital, Nizhny Novgorod, Russia; ^13^ Immunology Department, First St. Petersburg State Medical University named after acad. I.P. Pavlova, Saint Petersburg, Russia; ^14^ Immunology Department, Voronezh Regional Children’s Clinical Hospital, Voronezh, Russia; ^15^ Immunology Department, Republican Children’s Clinical Hospital named after E.P. Glinka, Chechen Republic, Russia; ^16^ Immunology Department, Regional Children’s Clinical Hospital, Republic of Buryatia, Russia; ^17^ Immunology Department, Republican Children’s Clinical Hospital, Republic of Crimea, Russia; ^18^ Immunology Department, Research Institute of Fundamental and Clinical Immunology, Novosibirsk, Russia; ^19^ Immunology Department, Children’s Republican Clinical Hospital, Kazan, Russia; ^20^ Immunology Department, Chelyabinsk Regional Children’s Clinical Hospital, Chelyabinsk, Russia; ^21^ Immunology Department, Children’s Regional Hospital, Perm, Russia; ^22^ Immunology Department, Regional Children’s Clinical Hospital named after N.N. Silishchevoy, Astrakhan, Russia; ^23^ Immunology Department, Regional Children’s Clinical Hospital No. 1, Tyumen, Russia; ^24^ Immunology Department, City Children’s Clinical Hospital №2 named after V.P. Bisyarina, Omsk, Russia; ^25^ Immunology Department, Irkutsk State Regional Children’s Clinical Hospital, Irkutsk, Russia; ^26^ Immunology Department, Children’s City Clinical Hospital, Kaluga, Russia

**Keywords:** inborn errors of immunity, subcutaneous immunoglobulin, intravenous immunoglobulin, efficacy, safety, tolerability, quality of life, rapid push

## Abstract

Subcutaneous immunoglobulin (SCIG) preparations are widely used in patients with inborn errors of immunity (IEI), with proven efficacy and good tolerance. We assessed treatment efficacy, safety, and quality of life in a large cohort of IEI patients who switched from intravenous immunoglobulin (IVIG) to SCIG. Our observational study included 200 patients aged 1–65 years with IEI. SCIG Cutaquig (16.5%) was administered every 7–10 days for at least 12 months via the rapid push method. We assessed the rate of infection, immunoglobulin G (IgG) concentration, adverse events, and quality of life. A total of 8,787 SCIG doses were administered during the study. The rate of infections (per person/month) during SCIG treatment was 0.05, which was significantly lower compared to 0.19 during the IVIG period (p<0.001). The median trough IgG was 6.9 g/L on IVIG, compared to 9.0 g/L during the first six months, and 9.2 g/L during the next six months on SCIG. Systemic reactions occurred in 12.4% of the IVIG infusions and 1.9% of the SCIG infusions. The total scores on quality of life summary assessments of physical and mental health were higher on SCIG therapy compared with IVIG (p<0.001). At the end of the study, 85.6% of the patients chose to remain on SCIG. Our data suggest that SCIG infusion via the rapid push method is effective, well tolerated, and feasible in large groups of IEI patients, including those in large countries such as Russia.

## Introduction

Inborn errors of immunity (IEI) is a group of genetic disorders that includes more than 550 different conditions (1). Immunoglobulin replacement therapy (IRT) using highly purified human immunoglobulin (IG) preparations is the standard of care for immunodeficiencies with impaired antibody production, which constitutes the majority of IEI (2). It is widely accepted that lifelong IRT substantially reduces the frequency and severity of infections by maintaining serum immunoglobulin G (IgG) concentrations close to physiological levels (3).

While the use of IG preparations for intravenous (IVIG) administration has been the most common type of IRT since the 1980s, subcutaneous immunoglobulin (SCIG) preparations are gaining popularity among physicians and patients due to their comparative ease of administration (4).

The conventional mode of SCIG administration uses a programmable infusion pump. More recently, subcutaneous push using a syringe and butterfly needle has emerged as an alternative and has been shown to be a comparable, if not a simpler and more convenient, method of administering SCIG (5). In countries where SCIG and infusion pumps are widely available, the choice of technique relies primarily on patient preference (5, 6).

SCIG preparations have not been easily accessible in Russia until recently. During the worldwide post-pandemic shortage of IVIG, 16.5% SCIG emerged as the preparation of choice for Russian IEI patients and presented an opportunity to assess real-life experience with SCIG delivered via the rapid push method in a large cohort of adult and pediatric patients with IEI.

Here we present the results of a multicenter observational study that evaluated the efficacy, safety, and tolerability of subcutaneous 16.5% immunoglobulin treatment as compared to previously used intravenous products.

## Materials and methods

### Study design and patients

Due to a plasma shortage following the COVID-19 pandemic, there was a significant shortage of IVIG supply in Russia. Beginning in 2022, 16.5% SCIG Cutaquig became available for all pediatric and some adult IEI patients due to a program of the nonprofit foundation Circle of Kindness, and a majority of IEI patients with antibody deficiencies were switched to SCIG. We conducted a prospective, non-interventional, open-label multicenter study of safety and feasibility with a retrospective phase (NCT05986734). The study subjects were recruited from patients who had been receiving IVIG treatment for at least six months prior and who had switched to SCIG treatment through the above-mentioned program. The main efficacy and safety parameters (see below) were assessed retrospectively for the six months of preceding IVIG treatment and prospectively for the first 12 months of SCIG treatment.

Pediatric and adult patients qualified for participation in the study if they had a documented diagnosis of IEI, required IgG replacement therapy, and had been receiving regular IVIG IRT for at least six months prior to inclusion in the study. Patients were ineligible if they had an active malignancy or had undergone hematopoietic stem cell transplantation (HSCT).

The study received Institutional Review Board approval, and prior to enrolling in the study, all patients or their legal guardians provided written informed consent.

The Russian IEI registry (7) was used as a platform for data collection, including the quality of life (QoL) questionnaires available to patients online through the registry.

A total of 233 IEI patients were originally enrolled in the study. During the prospective phase, 33 patients discontinued prematurely: 28 withdrew from the study but continued SCIG treatment, and five patients underwent HSCT. A total of 200 patients completed the study (**Supplementary Table 1**), and their data were analyzed. The median age of the patients was 11 years (1; 65), and the male/female ratio was 1.9:1. Patients were divided into the following age groups: <4 years, 4 to 7 years, 7 to 12 years, 12 to 18 years and over 18 years in accordance with the age groups of the quality of life questionnaires (PEDs-QL). (**Supplementary Figure 1**).

### Immunoglobulin treatment

Prior to recruitment in the study (Period 1), eligible patients had received IVIG every month at a median dose of  0.52 g/kg/month (min 0.3; max 0.89). During SCIG treatment (Period 2), patients received SCIG 16.5% (Cutaquig 16.5%, Octapharma, Switzerland) at the same monthly dose as in Period 1, divided into 3–4 infusions per month. The SCIG was infused using the rapid push method and 21G–26G needles. The product was administered at one or multiple injection sites at mean estimated rates of about 1.5 mL/min. All patients or their caregivers received training in SCIG administration techniques, and the first SCIG infusions were performed under the supervision of a trained nurse or physician.

A total of 8,787 SCIG infusions were administered during the study, 90.3% of which were at home.

The mean single dose was 0,14 g/kg (min 0.05, max 0.33) in 1–6 month of therapy and 0,15 g/kg (min 0,1, max 0,4) in 7–12 month of therapy.

The mean single injection volume was 17.12 mL (min 6; max 48) in 1–6 month of therapy and 17,68 mL (min 4,5, max 60) in 7–12 month of therapy. The duration of administration was 31,78 min (min 6, max 120) min in 1–6 month of therapy and 27,67 min (min 2, max 120) in 7–12 month of therapy (**Supplementary Table 2**).

### Efficacy assessment

The primary end-point was the rate of validated acute infections requiring additional antibiotic treatment, expressed as the average per person per month. Secondary end-points included frequency of hospitalizations, duration of inpatient hospital stays, days missed from school or work due to illness or infection, and duration of additional treatment with antibiotics. Trough levels of serum IgG were measured in serum samples taken immediately prior to immunoglobulin infusions at least three times during the specified IVIG and SCIG treatment periods.

### Safety

All adverse events (AEs) during IVIG and SCIG therapy were recorded. The investigator provided guidance to the patient or caregiver on how to identify and document local and systemic AEs.

### Measures of patient experience

QoL was surveyed in patients aged 2–18 years using the Pediatric Quality of Life Inventory Russian, version 4.0 (PEDS‐QL) questionnaire and in patients older than 18 years using the SF‐36 survey. On all questionnaires, higher scores indicated higher satisfaction.

The treatment burden related to IG therapy was evaluated using a special questionnaire developed within the framework of this study (**Supplementary Table 3**).

The severity of pain was assessed using the Wong–Baker scale (8).

### Statistical methods

Statistical analysis was performed using Microsoft Excel 2019 and RStudio Server 1.3.959. Quantitative indices were described using the median, first and third quartiles, minimum, and maximum. The calculated data were described in absolute values and percentages. Null observations were described using averages and fraction of zero values. The significance of differences between patient indices at different stages of the study was established using the Mann–Whitney test for paired data with the Bonferroni–Holm correction, where necessary. The significance of the differences among the data was determined using the chi-square test.

In data sets where most of the values equaled zero, average values were given without standard error of mean deviations. The p values equal to or less than 0.05 were considered significant.

## Results

### Serum trough IgG levels

**Figure 1:**
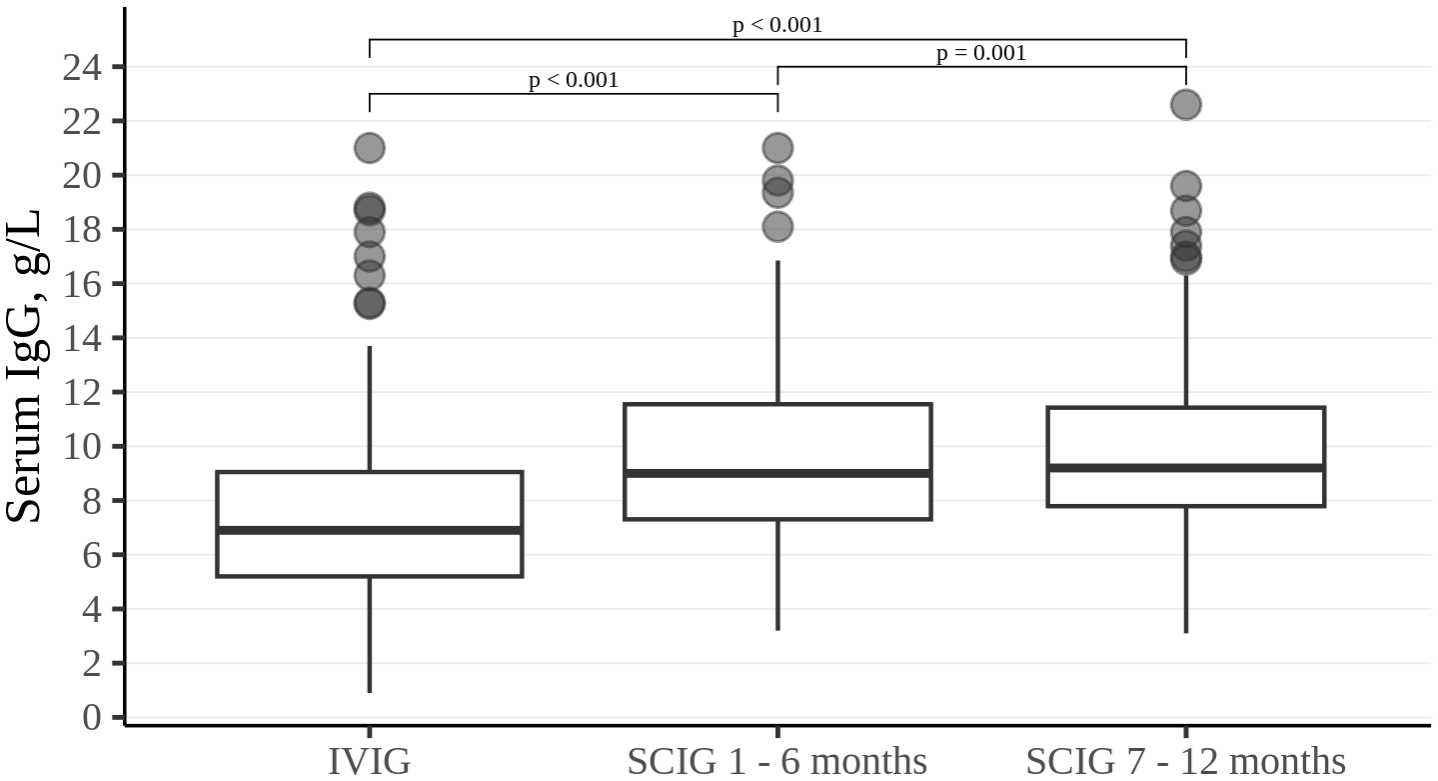
Trough IgG level in the study cohort. Thick lines represent median, boxes - the first and third quartiles.

Trough levels of serum IgG were higher during SCIG therapy than during IVIG therapy (p<0.001). Serum IgG values measured for six consecutive months of IVIG treatment attained a median of 6.9 g/L (5.2; 9.1). During the first six months of SCIG treatment, the median serum IgG level was 9 g/L (7.3; 11.6). At the end of the SCIG treatment study period, the median serum IgG levels reached 9.2 g/L (7.8; 11.4) (**Figure 1**).

### Efficacy

**Figure 2:**
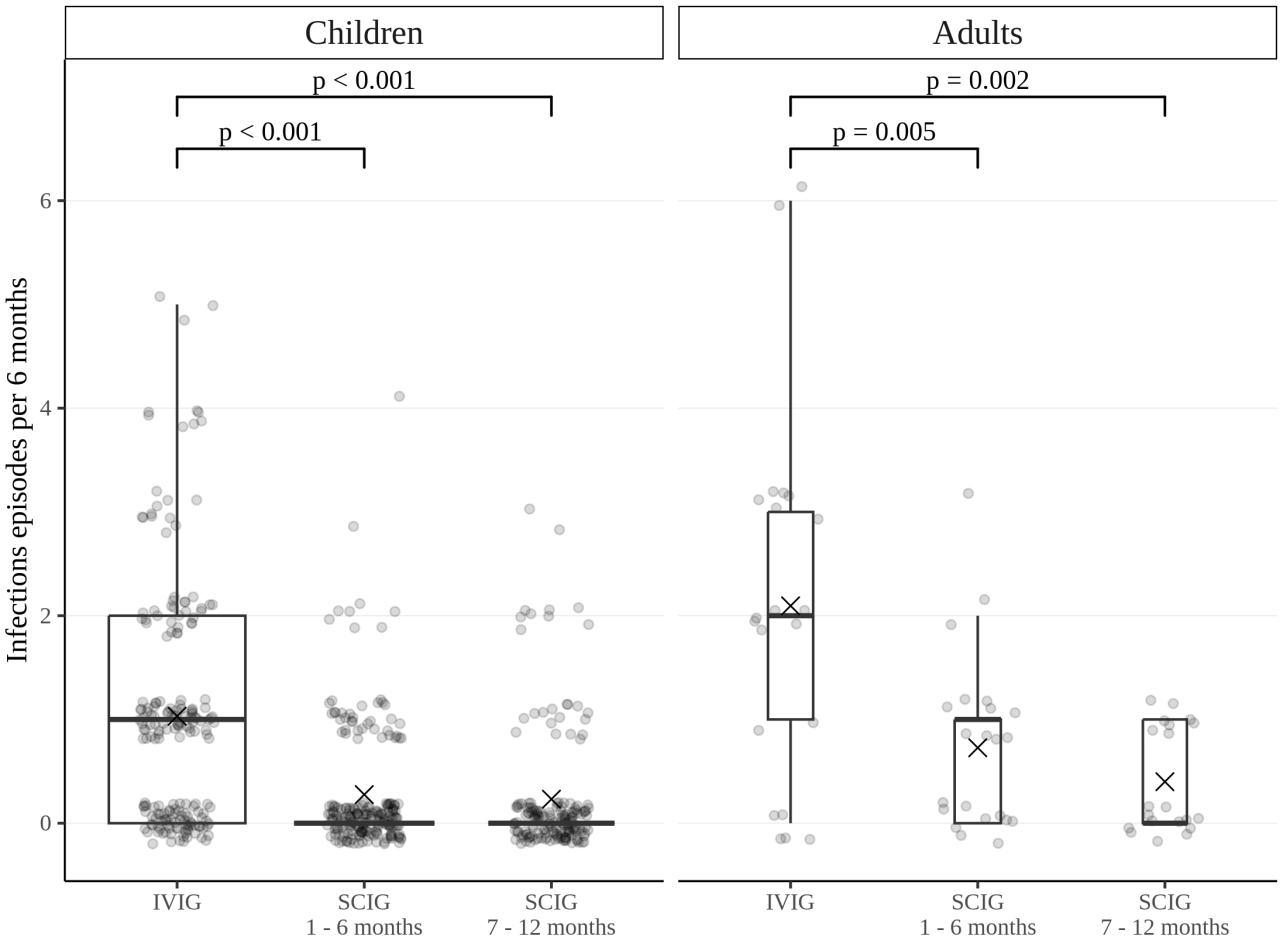
Frequency of infections in pediatric and adult patients. Infection rates are expressed as the number of episodes per six months. Thick lines represent the median, crosses represent the average, and boxes represent the first and third quartiles.

**Figure 3:**
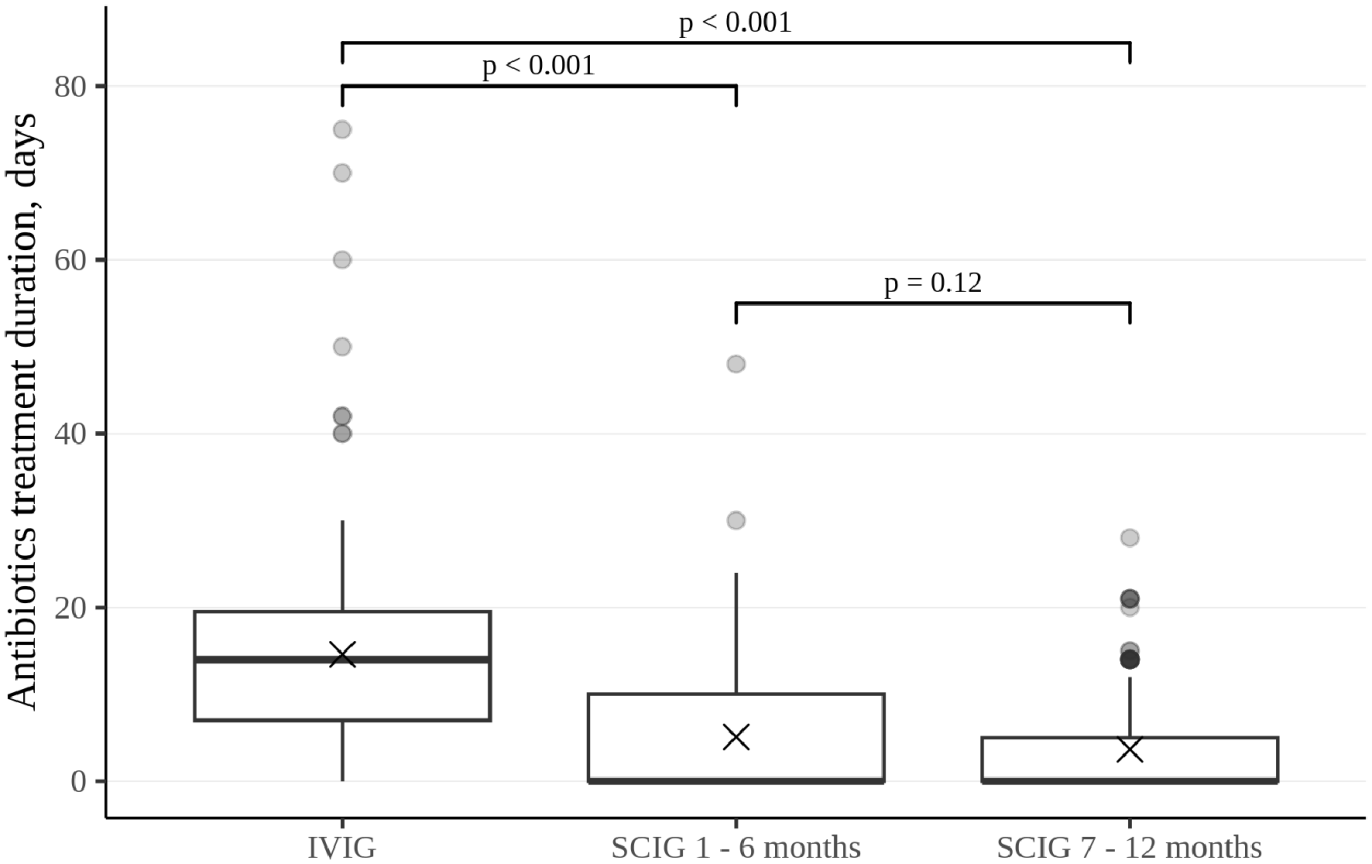
Duration of antibiotic treatment in patients who require it, presented as the total number of days per patient per each six-month interval. Thick lines represent the median, crosses represent the average, and boxes represent the first and third quartiles.

**Figure 4:**
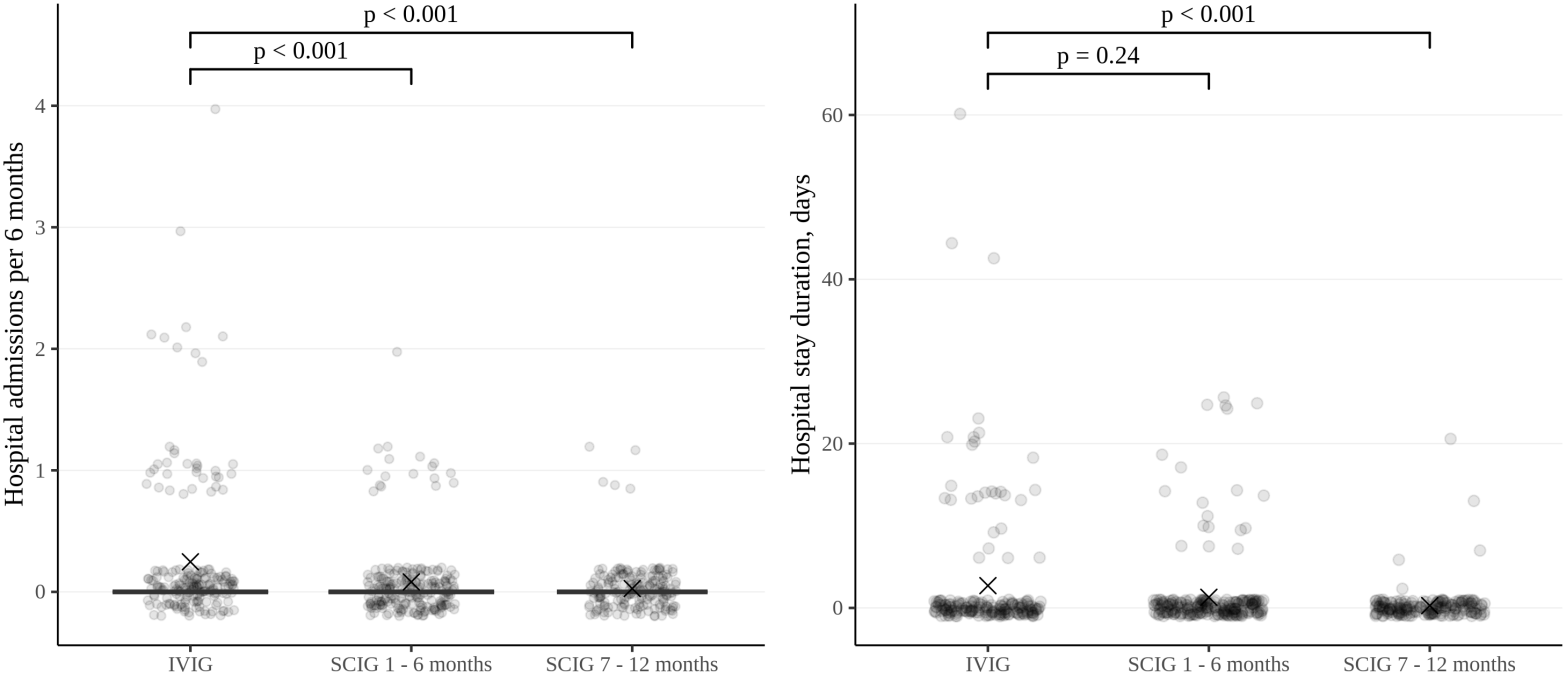
Frequency and duration of inpatient treatment of patients in the study cohort, expressed per patient per six-month period. Thick lines represent the median, crosses represent the average, and boxes represent the first and third quartiles.

The rate of infections during SCIG treatment was significantly lower than during IVIG treatment in adults and children (p<0.05) (**Figure 2**). The rate of infections during the entire SCIG treatment period was 0.05 per person/month, and 66% of the patients had no infections. The rate of infections during IVIG treatment was 0.19 per person/month, and 38% of the patients had no infections. Antibiotics used to treat infections were administered for a median of 1.53 days per person/month on IVIG, 0.49 days per person/month during the first six months of SCIG treatment, and 0.61 days per person/month during the next six months of SCIG treatment (p<0.001) (**Figure 3**). The median rates of hospitalization were 0.04 events per person/month during IVIG treatment and 0.01 events per person/month during SCIG treatment (p<0.001) (**Figure 4**). The durations of hospitalization were 0.44 days per person/month during IVIG treatment, 0.32 days per person/month during the first six months of SCIG treatment, and 0.045 days per person/month during the next six months of SCIG treatment (p<0.001) (**Figure 4**).

While receiving SCIG treatment, patients missed school or work an average of 2.94 days per month as compared to 3.66 days per month during IVIG treatment (p<0.05).

### Adverse events

**Figure 5:**
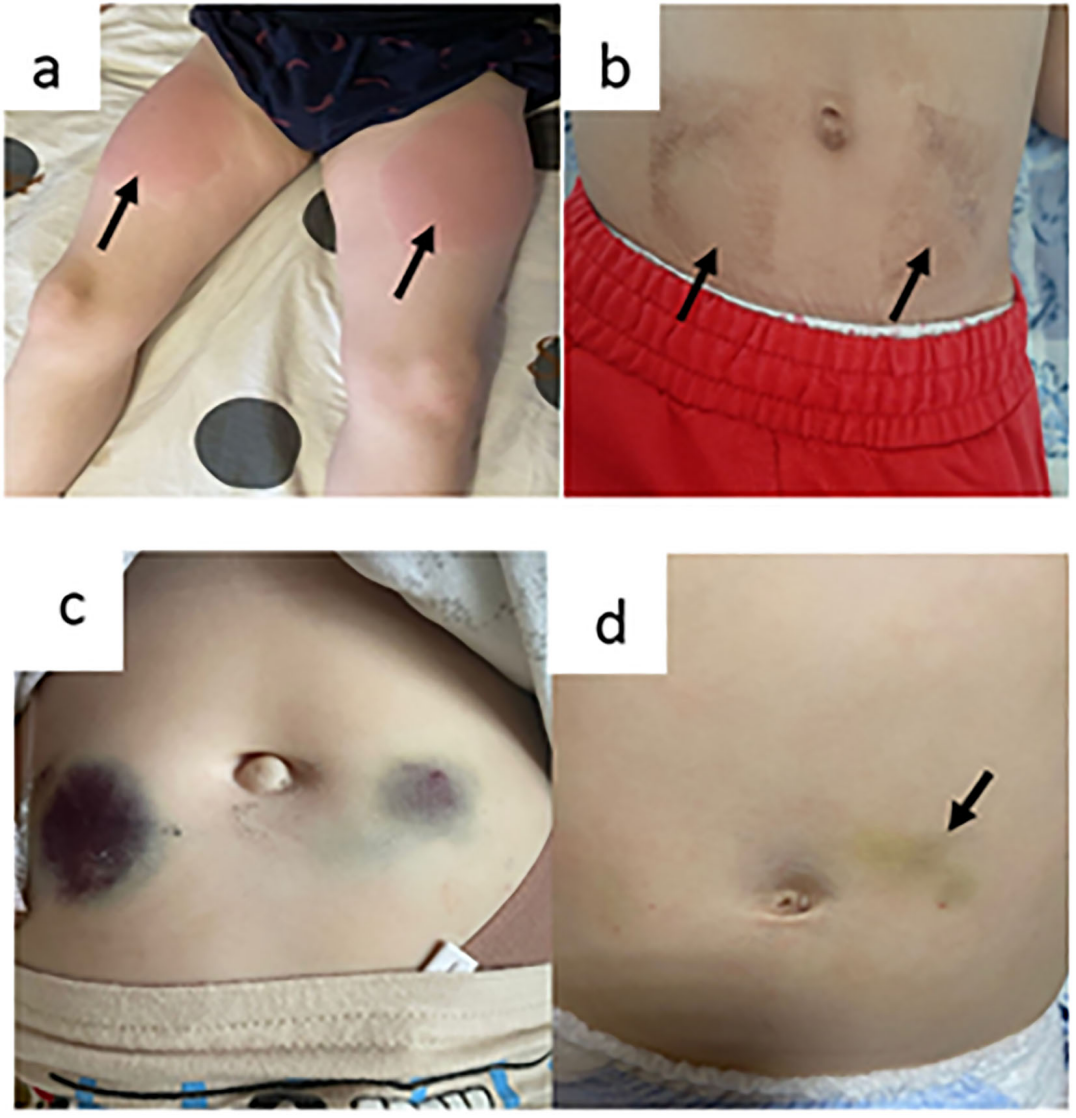
Local adverse events (**a**, **b** = contact dermatitis as a result of using an aseptic patch; **c** = hemorrhagic syndrome in a patient with a platelet count of 30,000 cells/µL; and **d** = hemorrhagic syndrome in patients with platelet counts of 3000 cells/µL).

Local reactions were the most common AE during SCIG treatment (**Table 1**). The development of expected local reactions accompanied about 70% of infusions of SCIG over 12 months of treatment, and none led to discontinuation of treatment. The incidence of infusion site reactions decreased over time. The majority of these reactions were mild or moderate, and most were of short duration. In three patients, we documented the development of contact dermatitis related to the use of an aseptic patch to secure a needle at the SCIG injection site (**Figures 5a, b**).

**Table 1:** Local reactions during SCIG treatment.

Type of reaction	SCIG 1–6 months, number (%) of infusions	SCIG 7–12 months, number (%) of infusions
No reaction	1266 (28.2%)	1620 (36.9%)
Infiltration + hyperemia of the injection site lasting less than 4 hours	1800 (40%)	1788 (40.7%)
Infiltration + hyperemia of the injection site lasting 5 to 12 hours	723 (16%)	734 (16.7%)
Infiltration + hyperemia of the injection site lasting 12 hours to several days	276 (6.1%)	168 (3.8%)
Itching	96 (2.1%)	–
No data	334 (7.4%)	73 (1.6%)

During the study, 7 (3.5%) patients (1.93% of infusions) experienced systemic reactions to SCIG, and none led to discontinuation of treatment. In addition, 39 (19.5%) patients (12.4% of infusions) experienced systemic reactions to IVIG (p<0.001). The most common systemic adverse reactions to IVIG were fever, headache, lower back/leg pain, asthenia, vomiting, seizures, hypertension, rash (**Table 2**).

**Table 2:** Systemic adverse events in the study cohort.

Reaction	6 months of IVIG, episodes (percent of total number of infusions)	SCIG, episodes (percent of total number of infusions)	
		1–6 months	7–12 months
Fever	57 (4.07%)	1 (0.02%)	–
Headache	37 (2.64%)	13 (0.31%)	47(1.07%)
Lower back/leg pain	27 (1.93%)	–	–
Asthenia	27 (1.93%)	–	1 (0.02%)
Vomiting	9 (0.64%)	15 (0.33%)	6 (0.14%)
Seizures	3 (0.21%)	–	–
Hypertension	8 (0.57%)	1 (0.02%)	–
Rash	6 (0.42%)	–	–
Total	174 (12.41%)	30 (0.7%)	54(1.23%)

### SCIG treatment in special cohorts of patients

A total of 210 SCIG infusions were performed in eight patients with immune/other thrombocytopenia and the following IEI: Wiskott–Aldrich syndrome, 4; STAT1 gain-of-function defect, 2; LRBA defect, 1; and combined immunodeficiency without genetic verification, 1. The median platelet count in this patient group was 39,000 cells/µL (min 3000; max 130,000). The severity of skin hemorrhagic syndrome was correlated with platelet count. There was no cutaneous hemorrhagic syndrome in patients with platelet counts of 30,000 cells/µL or more (**Figure 5c**). In one patient with a platelet count of 3000 cells/µL, a hematoma of up to 4 cm in diameter developed at the injection site (**Figure 5d**).

In all, 278 infusions of SCIG were performed in patients with severe dermatitis: 134 in three patients with generalized specific dermatitis within the framework of Netherton syndrome and 144 infusions in four patients with atopic dermatitis (two with hyper-IgE syndrome and two with Wiskott–Aldrich syndrome). Local reactions in this group did not differ from those in the general cohort. Specifically, no infections at injection sites or worsening dermatitis were noted.

### Patient experiences

In patients under 18 years of age, improvement in quality of life was observed in all age subgroups. Overall, on the pediatric cohort’s questionnaire, the median total score was 900 (0; 1475) at the end of the IVIG treatment period and 1075 (225; 1500) at the end of 12 months of SCIG treatment (p<0.001). On the parents’ questionnaire, the median summary score at the end of the IVIG treatment was 875 (0; 1475) and 1200 (100; 1500) at the end of 12 months of SCIG treatment (p<0.001) (**Supplementary Table 4**).

Interestingly, in adult patients, no significant changes in the quality of life were recorded between the IVIG and SCIG treatment periods (p>0.05), which can be explained by the small number of patients over age 18. At the end of IVIG treatment, the median score on the mental component was 35.67 (26.91; 50.69) and on the physical component, 44.93 (33.49; 62.04). After 12 months of SCIG treatment, the median score on the mental component was 42.18 (25.6; 51.26) and on the physical component, 48.73 (29.68; 62.11) (**Supplementary Table 5**).

**Figure 6:**
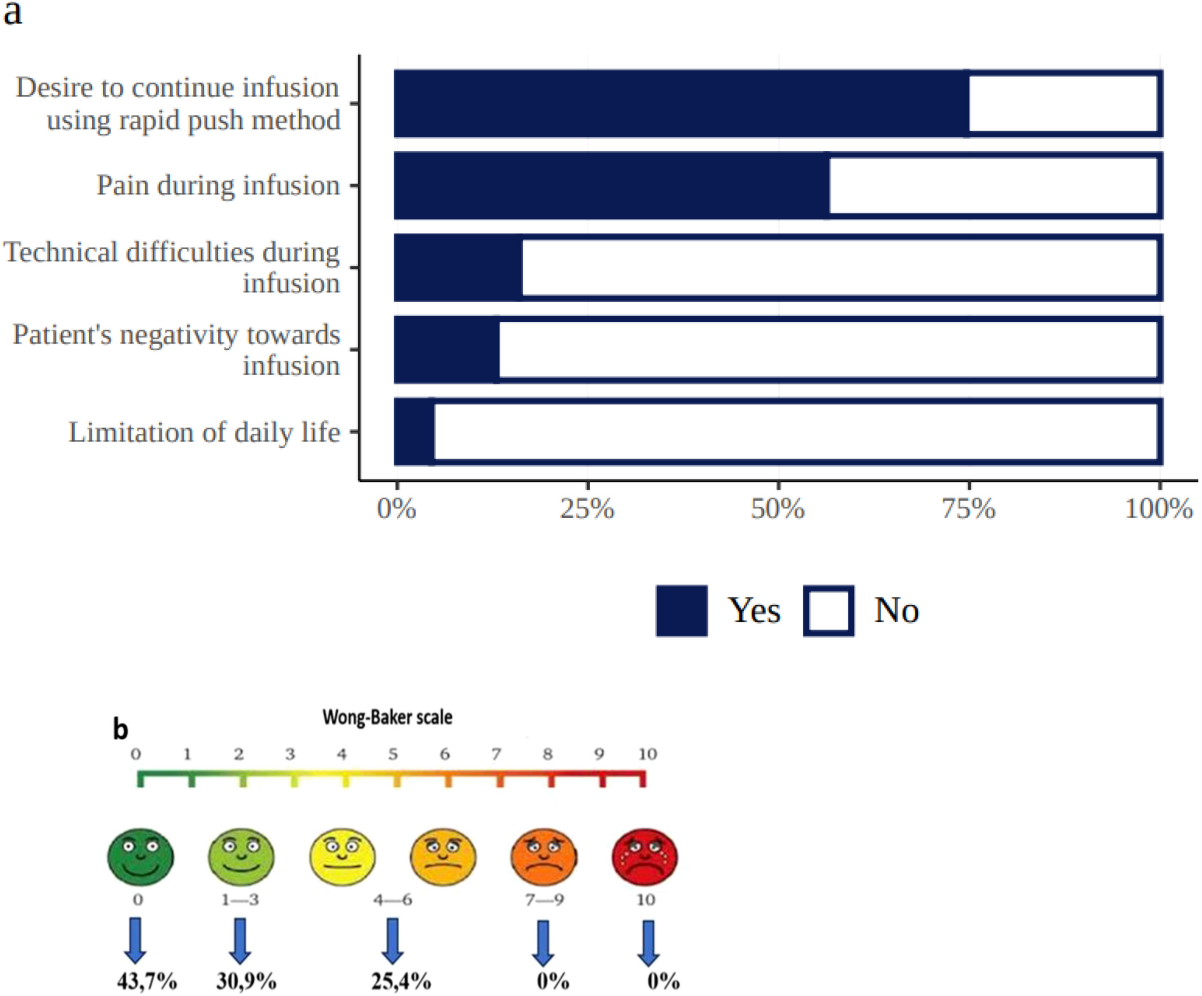
Patient experiences during SCIG treatment (**a** = treatment-related burden, **b** = Wong–Baker scale, demonstrating pain level distribution in 112 patients who experienced pain).

At the end of the study, 83% of patients reported that they preferred SCIG self-infusions to IVIG. The remaining patients either had no preference or preferred IVIG therapy. According to the survey, 74% of patients wanted to continue the infusions using the rapid push method, which caused no technical difficulties in 84% of respondents (**Figure 6a**). Although 56.3% of the patients experienced some pain during subcutaneous administration of the drug, the severity of this pain in most cases (74.6%) was mild and ranged from 0–3 on the Wong–Baker scale (**Figure 6b**). The retrospective nature of the study’s IVIG treatment phase did not allow for the accurate assessment of pain during intravenous infusions, but many parents of the pediatric patients reported it to be significant.

## Discussion

This observational study is the first large-scale experience with SCIG in Russia, where more than 600 IEI patients currently receive SCIG, and 200 were recruited into and completed this study.

The results obtained with 16.5% SCIG substitution therapy in patients with IEI demonstrate efficacy and safety comparable or superior to that of the previously used IVIG.

SCIG at monthly doses equivalent to those of IVIG was more efficient in preventing infections, as previous studies have shown (9–11). During SCIG treatment in our study, the use of additional antibiotics to treat infections was low and comparable to the results obtained in other large studies (12, 13).

In our study infection frequency was 0.6 per patient/year. Previously published infection rates describe a yearly rate per patient of 5.18 (95% CI 4.305, 6.171) for IGSC 20% (14), 2.76 for IgPro20 (15), 3.95 for a 16% SCIG (11) and 4.1 for a 10% SCIG (12). Although it is difficult to compare efficacy results among studies due to differences in patient populations and study designs, infection rates observed in the our study are even lower than those reported for other SCIGs.

Another important parameter, the duration of antibiotics use is difficult to compare between the studies as practice of prophylactic antibiotics varies significantly between the countries and the patients’ groups. Our study demonstrates lower antibiotics use rate (12.12 days per person per year) in comparison with European centers (20.49 days per person per year) (16).

The retrospective nature of the IVIG treatment period assessment is a limitation of the present study due to the variability of the IVIG preparations used and the theoretical possibility of errors in recording the study’s parameters, yet the results and the outcomes of additional assessments (e.g., days missed from school or work, number and duration of hospitalizations) establish the high efficacy of SCIG 16.5% in the Russian cohort of IEI patients.

SCIG is administered every 3–14 days compared to every 21–28 days for IVIG. This method provides more consistent trough concentrations of serum IgG compared to the initial high peaks followed by low troughs that are associated with IVIG therapy (16, 17). In the present study, the median levels of serum IgG troughs remained above 8 g/L throughout the SCIG treatment, a result similar to that shown by Kobayashi et al. (16, 18) and one that explains the better control of the disease.

Compared to intravenous administration, subcutaneous administration of immunoglobulin is associated with a lower incidence of systemic side effects (18). Several studies have found similar low frequencies of systemic reactions to SCIG therapy in comparison with IVIG (19–21).

The incidence of systemic reactions to SCIG was also lower than reported by others (0.01 per infusion in our study vs 0.028 - 0.697 per infusion in meta-analysis by Orange et al) which yet again supports the fact that the majority of patients tolerate SCIG well (22).In the current study expected local AEs were mostly short-lived and easily managed by the patients. In addition, some local reactions at the injection site turned out to be unrelated to the injection or preparation but instead resulted from an allergic reaction to adhesive coverings used to cover the prick wound. Hence, it is important to treat atypical local reactions with this possibility in mind.

SCIG preparations have significant potential, particularly for Russian patients with IEI. In most countries, IVIG can be administered only in a hospital or outpatient clinic environment, although some centers do allow intravenous administration by nurses in the patient’s home. Russia is a vast country with some sparsely populated areas. As a result, many IEI patients must travel for many hours to reach an IVIG infusion clinic. This regular commute requires significant nonmedical expenses and time off from work or school, which in turn leads to decreased QoL and/or treatment compliance. In contrast, SCIG can be self-administered at home. As a result, SCIG administration does not interfere with daily activities and enables patients to maintain normal lifestyles.

Overall, patient‐centered outcomes showed that IEI patients preferred SCIG replacement therapy to IVIG, in line with the results of other studies (23–26). In the present study, 94% chose SCIG treatment to be conducted at home, and most were happy with the manual SCIG infusions via the rapid push method. Similarly, Shapiro showed that 71% of patients preferred the manual method compared to pump infusion (5).

In real-world practice, SCIG use in patients with thrombocytopenia of varying severity is important, yet underreported. The literature contains only very limited reports of SCIG injections in patients with thrombocytopenia (27). Pedini et al. safely used SCIG in three patients over the age of 18 with common variable immunodeficiency and immune thrombocytopenia (28). The present study demonstrates the safety of SCIG treatment—even in patients with severe thrombocytopenia—and a lack of skin AEs when platelet counts are higher than 30,000 cells/µL.

The present study also demonstrates the safety of using SCIG in patients with severe dermatitis. Skin lesions in patients with IEI are quite common, so these results are important.

In conclusion, the results of our study demonstrate that SCIG treatment was accompanied by a low frequency of infections and systemic adverse reactions and maintained protective trough levels of serum IgG. It also demonstrates the success of IRT in a hospital-free setting and a reduction in treatment-associated efforts. Importantly, it also demonstrates improvements in patients’ quality of life. The present study proves that it is possible to successfully switch from IVIG to SCIG in a large cohort of IEI patients in a country that has had very limited prior experience with SCIG therapy (29).

## Data Availability

The original contributions presented in the study are included in the article/**Supplementary Material**. Further inquiries can be directed to the corresponding author.

## Supplementary material

Supplementary Figure 1 Age groups of the patients in the study.

Supplementary Table 1 EI diagnosis.

Supplementary Table 2 SCIG infusion parameters.

Supplementary Table 3 Questionnaire for patients/parents of patients receiving regular immunoglobulin replacement therapy for subcutaneous administration.

Supplementary Table 4 The results of the PEDS‐QL survey.

Supplementary Table 5 The results of the SF-36 survey.
