# Supplementary material for: Рrospective multicenter study of treatment efficacy, safety, and quality of life in a large cohort of patients with inborn errors of immunity receiving subcutaneous immunoglobulin by the rapid push method
Source: Front Immunol. 2025 Jul 22;16:1598491. doi: 10.3389/fimmu.2025.1598491 (PMC12321879; doi:10.3389/fimmu.2025.1598491)
Supplement: Supplementary Figure 1 — Age groups of the patients in the study. [file SupplementaryFile1.pdf]

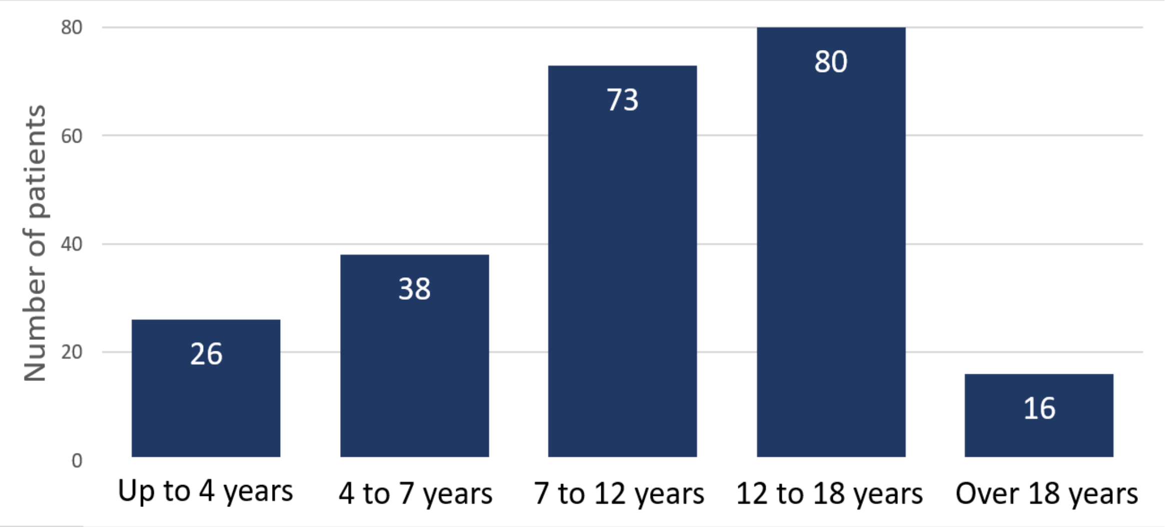

Figure 1. Age groups of the patients in the study.

Table 1. IEI diagnosis

Diagnosis	Number of patients
Combined immunodeficiencies associated with syndromic pathology	
Wiskott-Aldrich syndrome	6
Ataxia-telangiectasia	18
Ligase 4 deficiency (LIG4)	1
Nijmegen breakage syndrome	2
Centromeric region instability and facial anomalies syndrome (ICF)	1
DiGeorge syndrome	23
Jacobsen syndrome	2
Cartilage-hair hypoplasia (CHH)	2
Schimke immuno-osseous dysplasia	5
Hyper-IgE syndrome	7
Netherton syndrome	3
Kabuki syndrome	7
Roifman syndrome	1
Down syndrome	1
Primary immunodeficiency with predominant antibody deficiency	
X-linked agammaglobulinemia	44
Common variable immunodeficiency (CVID)	20
Activated phosphoinositide 3-kinase delta syndrome (APDS)	7
TRNT1 defect	1
Primary immunodeficiencies with immune dysregulation	
Autoimmune Polyendocrinopathy-Candidiasis-Ectodermal Dystrophy (APECED)	6
CTLA4 insufficiency	4
LRBA deficiency	4
STAT3 gain-of-function (GOF)	1
Autoimmune lymphoproliferative syndrome (ALPS)	1

Defects of innate immunity	
STAT1 gain-of-function (GOF)	7
NBAS defect	3
Autoinflammatory diseases	
Deficiency of adenosine deaminase-2 (ADA2),	2
Autoinflammation with phospholipase C gamma 2-associated antibody deficiency and immune dysregulation (APLAID)	2
Primary immunodeficiencies with defects in cellular and humoral immunity	
X-linked hyper IgM syndrome (HIGM1)	4
DOCK 8 deficiency	2
Combined immunodeficiency without specified genetic defect	46

Table 2. SCIG infusion parameters

Parameter	1–6 months of therapy	7–12 months of therapy
Single dose, g/kg	0,14 (min 0.05, max 0.33)	0,15 (min 0,1, max 0,4)
Single volume, mL	17,12 (min 6, max 48)	17,68 (min 4,5, max 60)
Duration of administration, min	31,78 (min 6, max 120)	27,67 (min 2, max 120)

Table 3. Questionnaire for patients/parents of patients receiving regular immunoglobulin replacement therapy for subcutaneous administration.

Variants of responses	Distribution of responses, % of respondents
1. Did you have any difficulties acquiring supplies (needles, syringes) for the "rapid push" immunoglobulin infusion?	
Yes, due to high cost	12.7%
Yes, due to the issues with availability or delivery	18.6%
No	65.7%
Other	2.85%
2. Where do you prefer to perform subcutaneous immunoglobulin infusion to yourself/your child?	
At home	94.2%
In-patient hospital stay	2.8%
In an outpatient clinic	2.8%
3. Did you have any technical difficulties during the subcutaneous immunoglobulin infusion to yourself/your child?	
Yes (please fill 4)	7%
I'm not sure	8.6%
No	84%
4. What technical difficulties did you have during the subcutaneous immunoglobulin infusion to yourself/your child?	
Issues with placement of the infusion needle	26.9%
Manual injection requires too much physical effort	42.3%
Patient’s anxiety	26.9%
Other	3.8%

5. Would you like to continue the infusion of subcutaneous immunoglobulin via “rapid push” method?	
Yes, it is fast and convenient	74%
No, due to technical difficulties	7.9%
No, due to the negative emotional reactions of the child	10%
No, due to pain	7.9%
No, other reasons	-
6. How does the treatment with subcutaneous immunoglobulin affect your daily life?	
Seriously affects work/study schedule	2.9%
Somewhat affects work/study schedule	1.4%
It does not affect daily life in any way	95.6%
7. Does subcutaneous immunoglobulin therapy limit your ability to travel far from home?	
Yes	14.3%
No	85.7%
8. Did your child have any negative emotional reaction during subcutaneous immunoglobulin infusion?	
Yes, there is negativity during each infusion of the drug	12.9%
Sometimes	41.5%
No, my child treats the administration of the drug as a routine procedure	45.4%
9. In general, how would you characterize your health/health of your child during subcutaneous immunoglobulin therapy?	
Poor	13.7%
Satisfactory	18.7%
Good	55%
Very good	7.5%
Excellent	5%
10. Do you prefer replacement therapy with intravenous or subcutaneous immunoglobulin?	
Intravenous immunoglobulin	17%
Subcutaneous immunoglobulin	83%

Table 4. The results of the PEDS-QL survey.

	IVIG		SCIG 1-6 months		SCIG 7-12 months	
Median (min;max)						
	Children	Parents	Children	Parents	Children	Parents
Physical	300 (0-500)	300 (0-500)	375 (0-500)	375 (0-500)	450 (0-500)	425 (0-500)
Emotional	225 (0-400)	225 (0-400)	275 (50-400)	275 (50-400)	300 (50-425)	300 (50-400)
Social	225 (0-300)	225 (0-300)	275 (0-300)	275 (0-375)	300 (0-325)	300 (0-300)
School	150 (0-300)	150 (0-300)	175 (0-300)	175 (0-300)	200 (0-300)	200 (0-300)

Table 5. The results of the SF-36 survey.

	IVIG	SCIG 1-6 months	SCIG 7-12 months

Median (min;max)				
Physical component	Physical Functioning	75 (20;100)	85 (5;100)	85 (30;100)
	Role-Physical Functioning	25 (0;100)	25 (0;100)	100 (0;100)
	Bodily Pain	62 (31;100)	100 (41;100)	72 (31;100)
	General health	40 (15;95)	45 (15;90)	57 (15;95)
Mental component	Vitality	45 (25;95)	50 (20;85)	55 (25;100)
	Social functioning	75(25;100)	62(25;100)	75(25;100)
	Role-emotional	33 (0;67)	33 (0;100)	33 (0;17)
	Mental health	72 (31;92)	64(32;92)	72(28;92)
